# Inhibition of poly(ADP-ribose) polymerase 1 protects against acute myeloid leukemia by suppressing the myeloproliferative leukemia virus oncogene

**DOI:** 10.18632/oncotarget.4748

**Published:** 2015-07-25

**Authors:** Lingbo Wang, Weili Cai, Wei Zhang, Xueying Chen, Wenqian Dong, Dongqi Tang, Yun Zhang, Chunyan Ji, Mingxiang Zhang

**Affiliations:** ^1^ Department of Hematology, Qilu Hospital, Shandong University, Jinan, China; ^2^ The Key Laboratory of Cardiovascular Remodeling and Function Research, Chinese Ministry of Education and Chinese Ministry of Public Health, Qilu Hospital, Shandong University, Jinan, China; ^3^ Department of Cardiology, The Third Hospital of Jinan, Jinan, China

**Keywords:** PARP-1, MPL, acute myeloid leukemia, prognosis

## Abstract

An abnormal expression of poly(ADP-ribose) polymerase 1 (PARP-1) has been described in many tumors. PARP-1 promotes tumorigenesis and cancer progression by acting on different molecular pathways. PARP-1 inhibitors can be used with radiotherapy or chemotherapy to enhance the susceptibility of tumor cells to the treatment. However, the specific mechanism of PARP-1 in acute myeloid leukemia (AML) remains unknown. Our study showed that expression of PARP-1 was upregulated in AML patients. PARP-1 inhibition slowed AML cell proliferation, arrested the cell cycle, induced apoptosis *in vitro* and improved AML prognosis *in vivo*. Mechanistically, microarray assay of AML cells with loss of PARP-1 function revealed that the myeloproliferative leukemia virus oncogene (MPL) was significantly downregulated. In human AML samples, MPL expression was increased, and gain-of-function and loss-of-function analysis demonstrated that MPL promoted cell growth. Moreover, PARP-1 and MPL expression were positively correlated in AML samples, and their overexpression was associated with an unfavorable prognosis. Furthermore, PARP-1 and MPL consistently acted on Akt and ERK1/2 pathways, and the anti-proliferative and pro-apoptotic function observed with PARP-1 inhibition were reversed in part via MPL activation upon thrombopoietin stimulation or gene overexpression. These data highlight the important function of PARP-1 in the progression of AML, which suggest PARP-1 as a potential target for AML treatment.

## INTRODUCTION

Acute myeloid leukemia (AML) is a clonal disorder of the hematopoietic system and is characterized by malignant proliferation, differentiation blockage, and unregulated apoptosis of hematopoietic stem progenitor cells (HSPCs). The incidence of AML is increasing worldwide while its etiology is still unclear. Increasing genetic and molecular abnormities have been proven to contribute to carcinogenesis, so diagnosis, risk stratification, and treatments targeting aberrant molecules and pathways are of clinical importance [1]. Although great improvements have been made in targeted therapies, many AML patients tend to have a poor prognosis because of marked heterogeneity of leukemic cells. There is an urgent need for the identification of new biomarkers and molecular mechanisms involved in AML progression for both fundamental research and clinical treatments.

PARP-1 is a nuclear protein, mainly known for DNA repair by catalyzing poly ADP-ribosylation of itself and other chromatin-associated proteins [2]. Besides, PARP-1 is implicated in a broad variety of cellular functions, including gene transcriptional regulation, cell cycle progression, inflammation, energy metabolism, cell proliferation and death [3–5]. In recent years, PARP-1 has been found to be highly expressed in breast cancer [6], hepatocellular carcinoma [7], and nasopharyngeal carcinoma [8]. Moreover, its overexpression inversely correlates with the prognosis [9, 10]. Mechanistically, PARP-1 decreases sodium iodide symporter mRNA levels by changing its epigenetic status in thyroid cancer cells [11]. Furthermore, PARP-1 up-regulates vimentin expression in aggressive metastatic melanomas, promoting an endothelial to mesenchymal transition, tumor angiogenesis, and distant metastases [12]. Additionally, PARP-1 overexpression inhibits the recruitment of death-inducing signaling complex in pancreatic cancer cells, offering the ability to resist TNF-related apoptosis-inducing ligand (TRAIL) therapy [13]. It is now widely accepted that PARP-1 functions as an oncogene; however, its specific role in AML and precise molecular mechanisms have not been clarified.

The myeloproliferative leukemia virus oncogene (MPL) encodes the 635 amino acid CD110 protein, which consists of four functional domains and belongs to the hematopoietic receptor superfamily [14]. Its ligand thrombopoietin (TPO) supports hematopoietic stem cell maintenance and proliferation as well as megakaryopoiesis [15]. Mutations in MPL or its secondary signaling proteins result in hyperproliferation of numerous cell lineages, causing hematopoietic diseases [16]. In AML, more specific signaling pathways related to MPL remain to be discovered.

In this study, we clarified the anti-proliferative and pro-apoptotic effects of PARP-1 inhibition in AML. As a monotherapy, PARP-1 inhibition alleviated the disease progression and prolonged survival in a mouse model of AML. Moreover, RNA microarrays of loss of PARP-1 function identified a significant downregulation of MPL. Further investigations revealed a significant positive correlation between PARP-1 and MPL overexpression in AML samples. Both PARP-1 and MPL overexpression was associated with an unfavorable prognosis. Finally, phosphorylation of Akt and ERK1/2 suggest that PARP-1 may be involved in these signaling pathways.

## RESULTS

### PARP-1 inhibition suppresses AML cell proliferation

**Figure 1:**
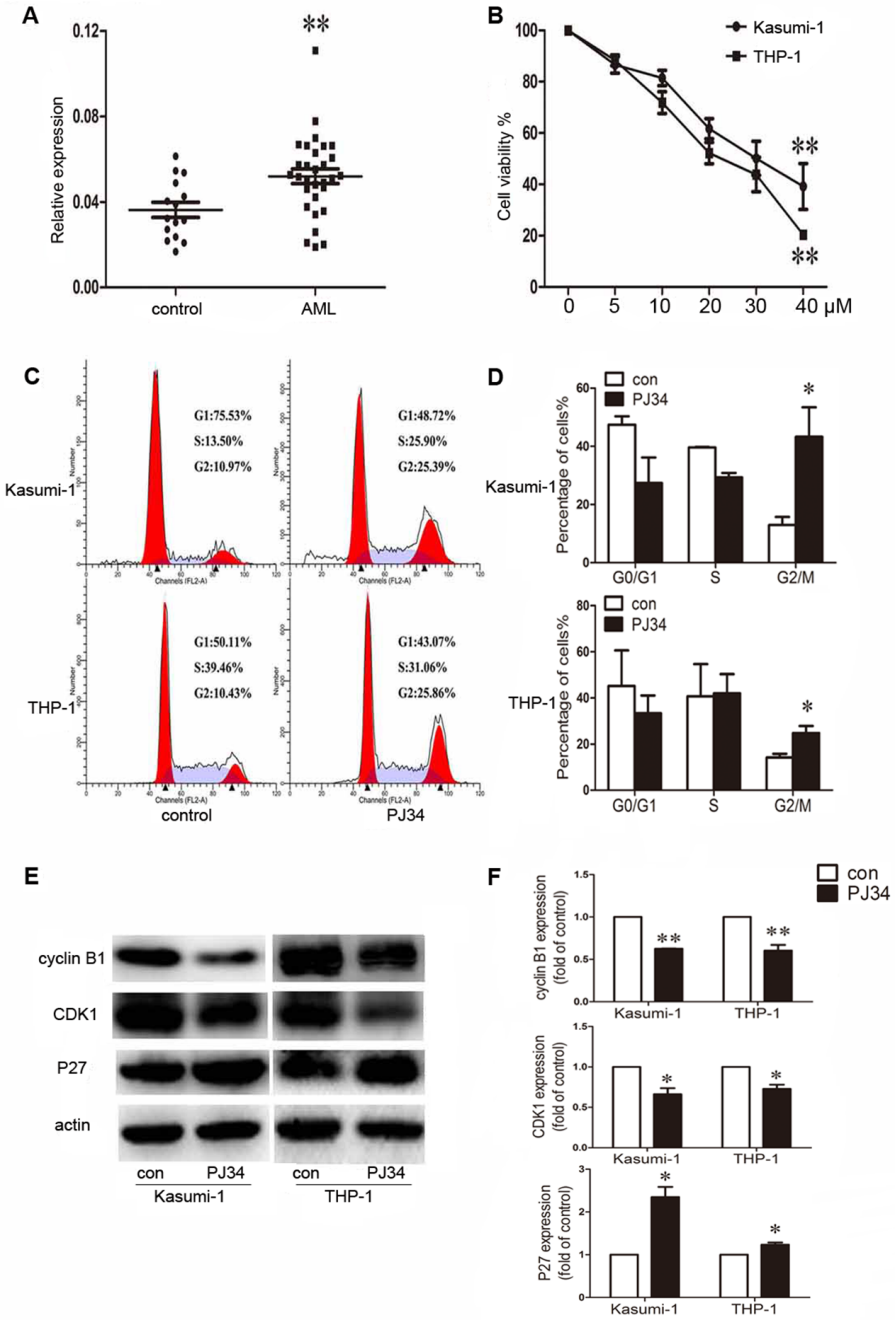
Aberrant expression of poly(ADP-ribose) polymerase 1 (PARP-1) in acute myeloid leukemia (AML) patients and effect of PARP-1 inhibition on proliferation and cell cycle in AML cell lines **A.** qRT-PCR analysis of PARP-1 mRNA in bone marrow from AML patients (*n* = 30) and controls (*n* = 15). Each point represents one sample. Horizontal bars represent the means, the whiskers represent SEM. **P < 0.01, AML vs. control. **B.** Cell viability of Kasumi-1 and THP-1 cells treated with 0, 5, 10, 20, 30, or 40 μM PARP-1 inhibitor PJ34. **P < 0.01, 40 vs. 0 μM. **C.** Flow cytometry and **D.** cell cycle quantification of AML cell G2/M arrest with PARP-1 inhibition. *P < 0.05, PJ34 vs. control. **E.** Western blot analysis of cyclin B1, CDK1, and P27 expression with PARP-1 inhibitor PJ34 or control treatment and **F.** quantification. *P < 0.05 and **P < 0.01, PJ34 vs. control.

In this study, we first detected the expression of PARP-1 in BM mononuclear cells from 30 AML patients, with 15 healthy donors as controls. PARP-1 expression was significantly increased in AML samples as compared to controls (*P* < 0.01; Fig. 1A).

**Figure 2:**
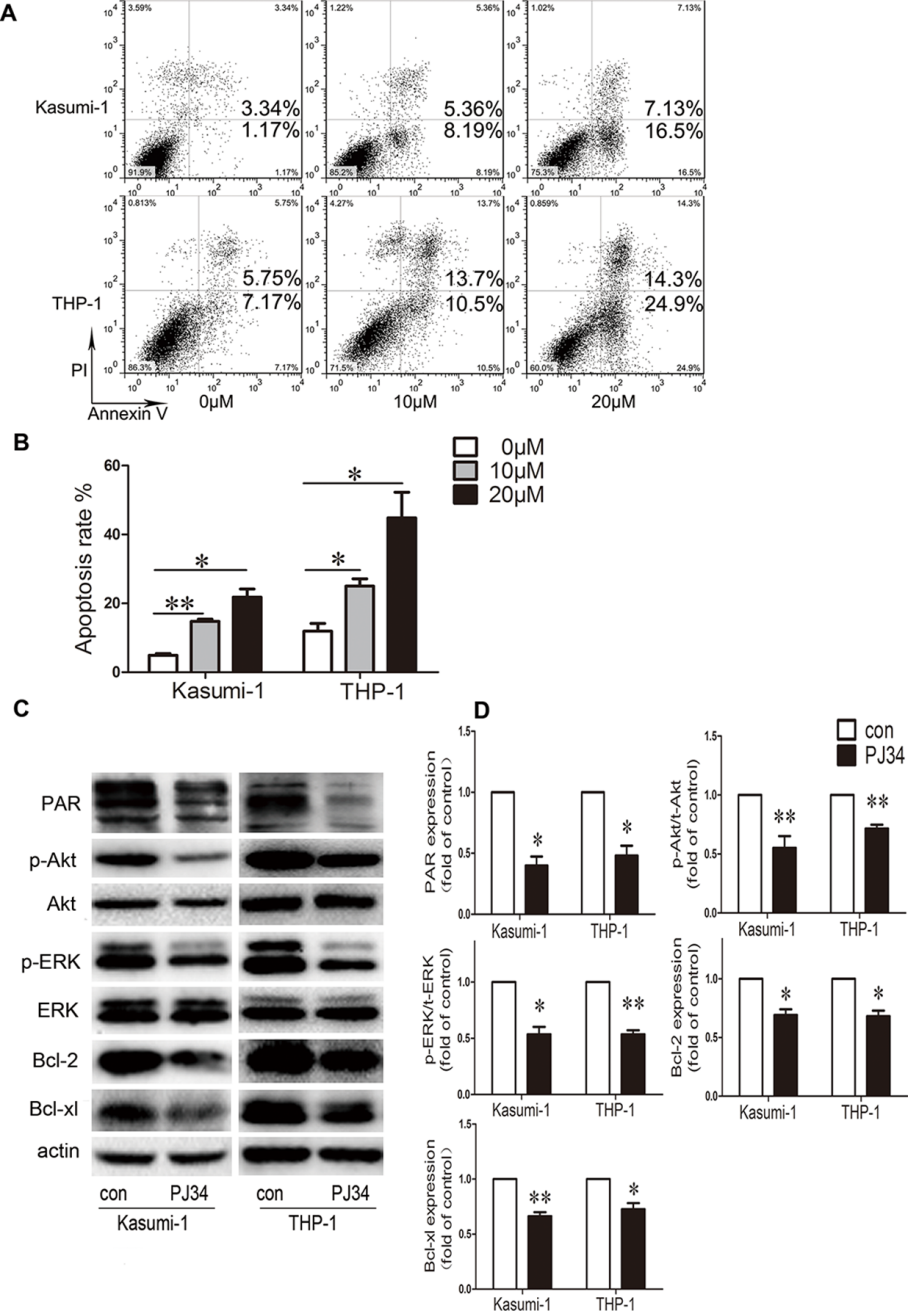
Effect of PARP-1 inhibition on apoptosis and molecular pathways in AML cell lines **A.** Flow cytometry and **B.** quantification of apoptotic AML cells stained with Annexin V-FITC and PI. *P < 0.05 and **P < 0.01, compared to 0 μM. **C.** Western blot analysis and **D.** quantification of PAR, p-Akt, t-Akt, p-ERK, t-ERK, Bcl-2, and Bcl-xL expression. *P < 0.05 and **P < 0.01, PJ34 vs. control. Data represent the mean ± SEM.

We determined the role of PARP-1 on growth of Kasumi-1 and THP-1 AML cell lines by CCK8 assay. PARP-1 inhibition with 5, 10, 20, 30, and 40 μM PJ34 dose-dependently decreased cell viability (*P* < 0.01; Fig. 1B). The half-maximal inhibitory concentration (IC_50_) of PARP-1 inhibitor PJ34 on Kasumi-1 and THP-1 cells was 23.5 ± 3.9 and 35.6 ± 5.5 μM, respectively. To ensure the specificity of the inhibition, we verified the growth inhibition effect on AML cell lines by PARP-1 gene interference (Supplementary Fig. 1). The results were consistent with those obtained with PARP-1 inhibitor PJ34. In exploring the underlying mechanism of PARP-1 inhibition in AML cells, we found a significantly higher number of cells arrested in the G2/M cell-cycle phase, and a decreased number of cells in the G0/G1 and S phases, with PARP-1 inhibition than without (Fig. 1C, 1D). Analysis of cell-cycle regulatory proteins showed decreased cyclin B1 and CDK1 levels accompanied by an increased P27 level in this process (Fig. 1E, 1F). As well, Annexin V-FITC/PI staining revealed an increased apoptosis of AML cells while increasing PARP-1 inhibition (Fig. 2A, 2B), which was further confirmed by lower levels of anti-apoptotic proteins Bcl-2 and Bcl-xL (Fig. 2C, 2D). These alterations might act on the Akt and ERK1/2 pathways because p-Akt and p-ERK levels were downregulated (Fig. 2C, 2D). Therefore, PARP-1 inhibition effect on AML cells is the result of both cell cycle arrest and apoptosis induction.

### PARP-1 inhibition significantly relieves leukemia progression in AML mice

**Figure 3:**
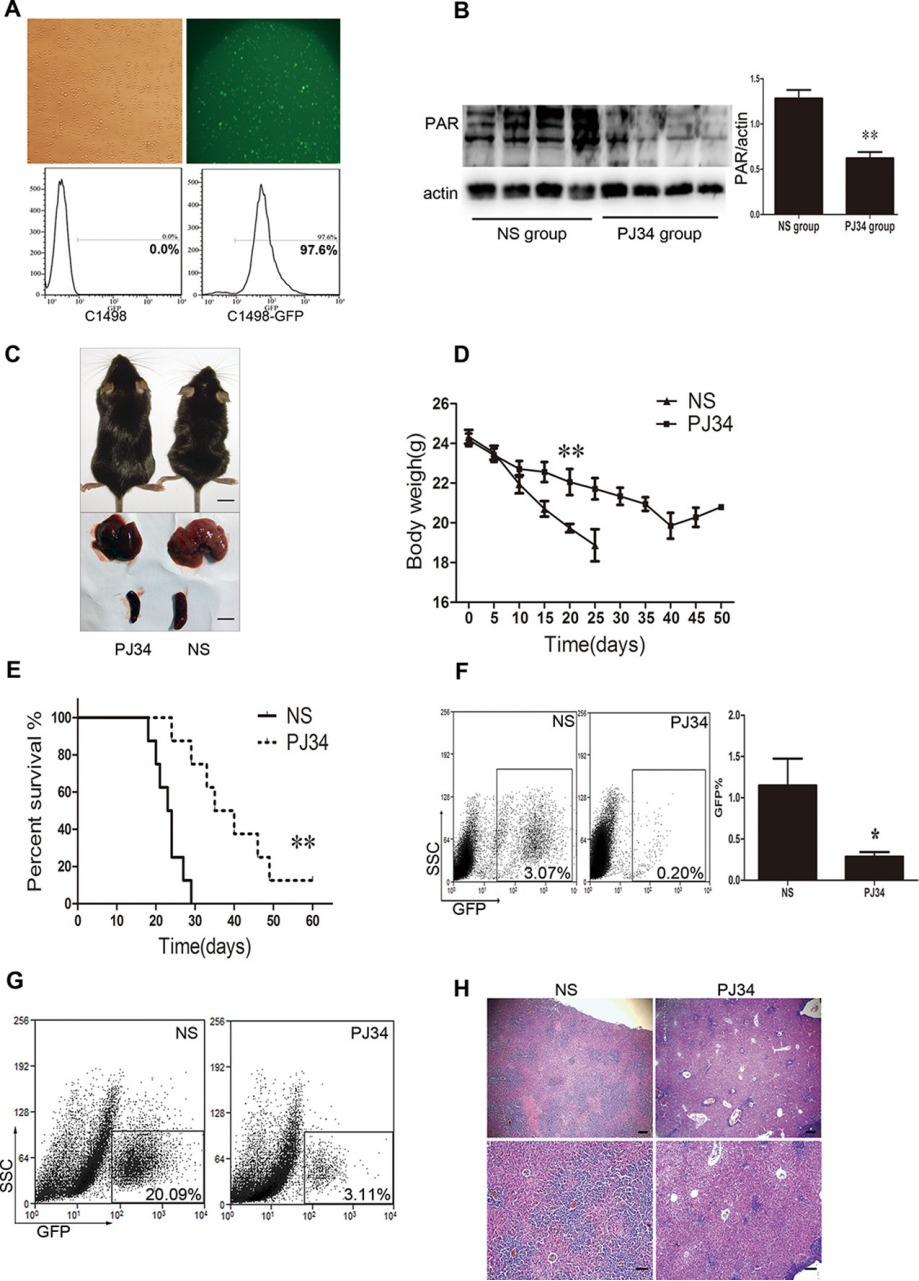
PARP-1 inhibition improves AML prognosis *in vivo* in AML mice **A.** Fluorescent microscopy and flow cytometry of C1498 cells transduced by a lentivirus with a GFP reporter. **B.** Western blot analysis of PAR expression in AML mouse tissue with and without PARP-1 inhibitor PJ34. **P < 0.01, PJ34 vs. normal saline (NS). **C.** Appearance, liver, and spleen of representative AML mice treated with and without PARP-1 inhibitor PJ34. Scale bar: 10 mm. **D.** Body weights of mice treated with and without PARP-1 inhibitor PJ34. **P < 0.01, PJ34 vs. NS. **E.** Survival of mice treated with and without PARP-1 inhibitor PJ34. **P < 0.01, PJ34 vs. NS. **F.** Flow cytometry analysis of GFP-positive cells in total peripheral blood leukocytes (*P < 0.05, PJ34 vs. NS) and **G.** liver monoplast suspension. **H.** Hematoxylin and eosin staining of hepatic tissues. Scale bars: 200 μm (top panels) and 100 μm (bottom panels). Data represent the mean ± SEM.

To test the function of PARP-1 in tumor progression *in vivo*, we used an AML murine model injected with C1498-GFP cells that were selected and maintained by puromycin and detected by flow cytometry (Fig. 3A). In this AML murine model, PARP-1 was inhibited by PARP-1 inhibitor PJ34 because of a reduced level of its enzymatic product, PAR (Fig. 3B). As expected, emaciation and weight loss were less severe with PJ34 than with saline injection (*P* < 0.01; Fig. 3C, 3D), and the median survival of AML mice treated with PARP-1 inhibitor PJ34 was prolonged as compared to control mice (37.5 vs. 23.5 days, *P* < 0.01; Fig. 3E).

The tumor burden was evaluated by organomegaly and tumor cell infiltration. Our results show that PARP-1 inhibition alleviated AML hepatomegaly and splenomegaly (Fig. 3C). Accordingly, the proportion of GFP-positive cells in the blood and liver tissues were significantly reduced with PARP-1 inhibition (Fig. 3F, 3G). Moreover, the number of leukemia cells was reduced in the liver (Fig. 3H). Therefore, PARP-1 inhibition alleviated AML tumor load *in vivo* in mice and prolonged their survival.

### PARP-1 inhibition downregulates MPL gene expression

**Figure 4:**
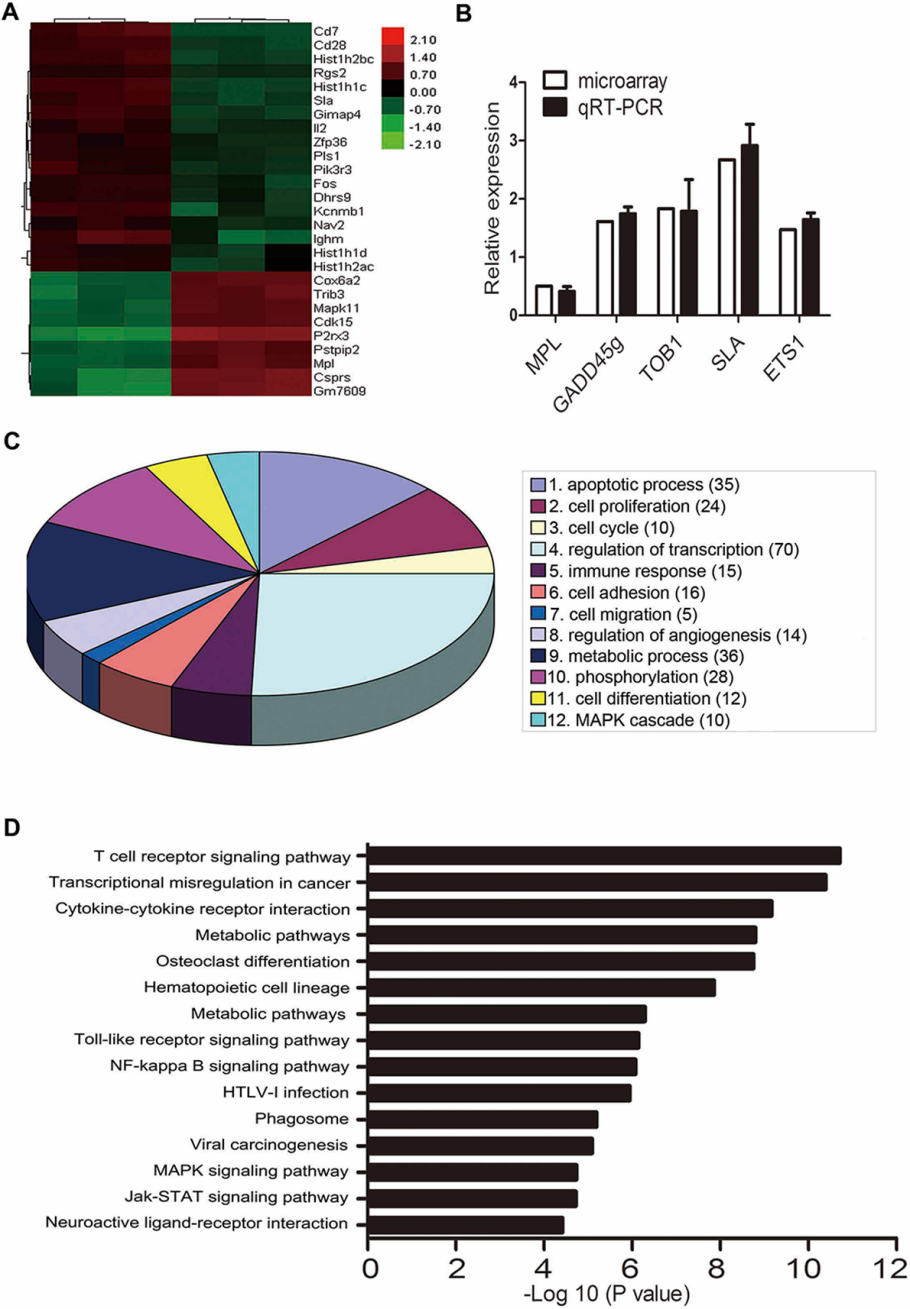
Microarray analysis of gene expression with PARP-1 inhibition **A.** Microarray assay of genes with >2.0-fold upregulation or ≥ 0.5-fold downregulation in C1498 mouse AML cells treated with PARP-1 inhibitor PJ34. **B.** qRT-PCR of 5 genes related to leukemia for validation of the microarray results. **C.** Gene ontology enrichment analysis of differentially expressed genes. The number of genes with a significantly changed expression is shown in parentheses. **D.** Top 15 enrichment pathways based on the KEGG database.

We performed a genome-wide microarray assay of C1498 cells with or without PARP-1 inhibition to profile differentially expressed genes that might be involved in PARP-1 regulation. As shown in Fig. 4A, we found 18 genes with >2.0-fold upregulation (Table 1) and 9 genes with ≥ 0.5-fold downregulation (Table 2) in 3 independent experiments (*P* < 0.01). To verify the microarray analysis data, we examined 5 differentially expressed genes that are involved in leukemia [17–21]. Similar results in gene expression were observed by qRT-PCR and microarray analysis with PARP-1 inhibition (*P* > 0.05; Fig. 4B). To understand some important biological processes affected by PARP-1 in AML cells, a GO analysis was performed and revealed PARP-1 roles in apoptosis, proliferation, cell cycle, cell differentiation, adhesion, migration, and angiogenesis (Fig. 4C). Pathway analysis with the KEGG database further classified the functional annotations of genes and revealed upregulated and downregulated genes significantly enriched in 86 pathways (15 top enrichment pathways are shown in Fig. 4D). Because microarrays showed a significant downregulation of the TPO receptor MPL with PARP-1 inhibition and MPL takes part in the biological behavior of leukemogenesis and chemoresistance [22–24], we focused on PARP-1 and MPL.

**Table 1:** Results of microarray analysis of the genes with over 2-fold upregulation following PARP-1 inhibition

Symbol	Official gene name	Probe ID	Fold change
*CD7*	CD7 antigen	10394054	3.15
*CD28*	CD28 antigen	10346783	2.72
*HIST1H1C*	histone cluster 1, h1c	10404059	2.87
*HIST1H2BC*	histone cluster 1, h2bc	10404053	2.74
*SLA*	src-like adaptor	10429128	2.67
*GIMAP4*	GTPase, IMAP family member 4	10538126	2.66
*RGS2*	regulator of G-protein signaling 2	10358389	2.2
*IL2*	interleukin 2	10497878	2.17
*FOS*	FBJ osteosarcoma oncogene	10397346	2.23
*NAV2*	neuron navigator 2	10553354	2.05
*DHRS9*	dehydrogenase/reductase (SDR family) member 9	10472538	2.14
*PLS1*	plastin 1 (I-isoform)	10595768	2.09
*KCNMB1*	potassium large conductance calcium-activated channel, subfamily M, beta member 1	10375137	2.7
*ZFP36*	zinc finger protein 36	10561453	2.09
*IGHM*	immunoglobulin heavy constant mu	10403069	3.18
*PIK3R3*	phosphatidylinositol 3 kinase, regulatory subunit, polypeptide 3 (p55)	10507273	2.3
*HIST1H1D*	histone cluster 1, h1d	10404033	2.04
*HIST1H2AC*	histone cluster 1, h2ac	10408220	2.2

**Table 2:** Results of microarray analysis of the genes with less than 2-fold downregulation following PARP-1 inhibition

Symbol	Official gene name	Probe ID	Fold change
*P2RX3*	purinergic receptor P2X, ligand-gated ion channel, 3	10484488	0.28
*COX6A2*	cytochrome c oxidase, subunit VI a, polypeptide 2	10568369	0.47
*TRIB3*	tribbles homolog 3 (Drosophila)	10488608	0.46
*MAPK11*	mitogen-activated protein kinase 11	10431410	0.48
*CDK15*	cyclin-dependent kinase 15	10346594	0.49
*MPL*	myeloproliferative leukemia virus oncogene	10515755	0.5
*PSTPIP2*	proline-serine-threonine phosphatase-interacting protein 2	10456904	0.5
*CSPRS*	component of Sp100-rs	10582879	0.36
*GM7609*	predicted pseudogene 7609	10347915	0.37

### MPL activation promotes the proliferation of AML cells

**Figure 5:**
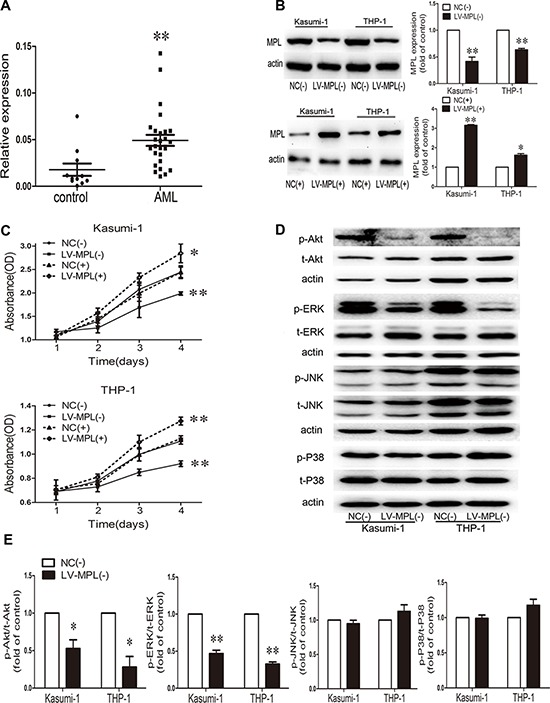
High expression of the myeloproliferative leukemia virus oncogene (MPL) in AML patients and MPL sustained malignant proliferation **A.** qRT-PCR analysis of MPL mRNA level in bone marrow from AML patients (*n* = 27) and controls (*n* = 11). Each point represents one sample. Horizontal bars represent the means, the whiskers represent SEM. **P < 0.01, AML vs. control. **B.** Western blot analysis and quantification of MPL protein expression by lentiviral infection in Kasumi-1 and THP-1 cells. NC(−), negative control of MPL knockdown; LV-MPL(−), MPL knockdown; NC(+), negative control of MPL overexpression; LV-MPL(+), MPL overexpression. *P < 0.05 and **P < 0.01, LV-MPL vs. NC. **C.** Cell viability of Kasumi-1 and THP-1 cells with MPL knockdown or overexpression. *P < 0.05 and **P < 0.01, LV-MPL vs. NC. **D.** Western blot analysis and **E.** quantification of p-Akt, t-Akt, p-ERK, t-ERK, p-JNK, t-JNK, p-P38, and t-P38 protein expression with MPL knockdown in Kasumi-1 and THP-1 cells. *P < 0.05, **P < 0.01, LV-MPL vs. NC. Data represent the mean ± SEM.

MPL is present on HSPCs, megakaryocytes, and thrombocytes [25–27]. To further clarify its role in AML, we first detected MPL mRNA in AML patient primary BM cells. MPL was significantly upregulated in AML samples compared to healthy control samples (*P* < 0.01; Fig. 5A). Further functional studies in Kasumi-1 and THP-1 AML cell lines of MPL knockdown or overexpression (Fig. 5B) showed a reduced or accelerated proliferation, respectively (*P* < 0.01; Fig. 5C). Therefore, MPL promotes AML cell malignant proliferation. Because MPL knockdown suppressed cell growth, we studied pathways that might be involved in this mechanism, including Akt and MAPK pathways. MPL knockdown notably decreased p-Akt and p-ERK levels (Fig. 5D, 5E) in both cell lines; however, no change in p-JNK and p-P38 levels was observed (Fig. 5D, 5E).

### PARP-1 inhibition protects against AML by downregulating MPL activity

**Figure 6:**
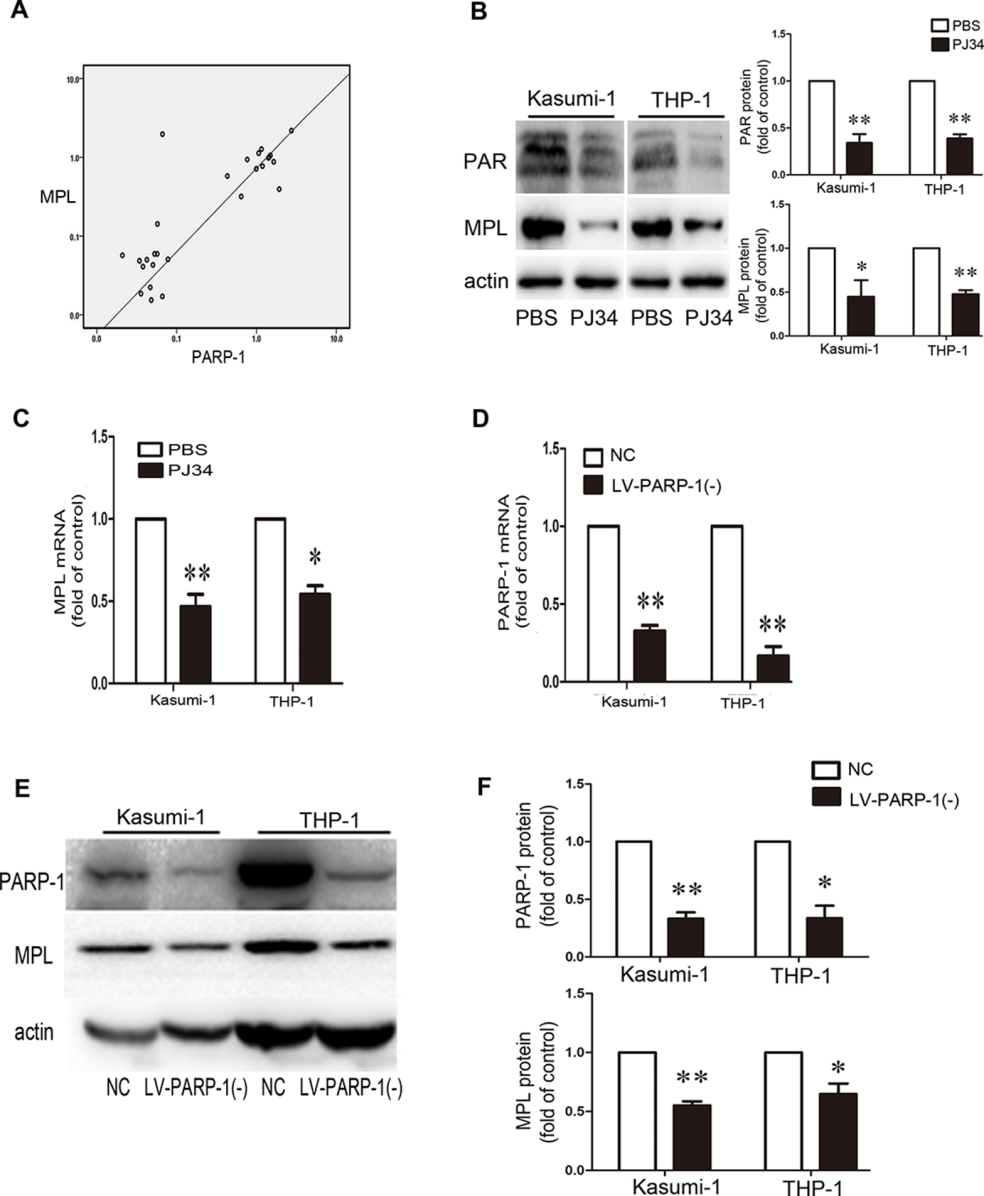
PARP-1 acts on MPL expression **A.** Positive correlation between PARP-1 and MPL expression in patient bone marrow samples (*n* = 27, *P* < 0.01). **B.** Western blot analysis of PAR and MPL protein levels and **C.** qRT-PCR analysis of MPL mRNA level with or without PARP-1 inhibitor PJ34. *P < 0.05, **P < 0.01, PJ34 vs. PBS. **D–F.** qRT-PCR and western blot analysis of PARP-1 expression upon lentivirus interference. NC, negative control of PARP-1 knockdown; LV-PARP-1(−), PARP-1 knockdown. *P < 0.05 and **P < 0.01, LV-PARP-1 vs. NC. **E, F.** Western blot analysis of MPL expression with PARP-1 gene silencing. *P < 0.05 and **P < 0.01, LV-PARP-1 vs. NC. Data represent the mean ± SEM.

According to our microarray results, MPL might be downstream of PARP-1. We compared MPL and PARP-1 expression in patient BM samples and we found that their expression positively correlated (*P* < 0.01; Fig. 6A). PARP-1 inhibition significantly inhibited PAR formation (Fig. 6B) and reduced the expression level of MPL (Fig. 6B, 6C). Further validation by PARP-1 knockdown (Fig. 6D–6F) showed that MPL protein levels were decreased (Fig. 6E, 6F). Therefore, PARP-1 may directly act on MPL expression.

**Figure 7:**
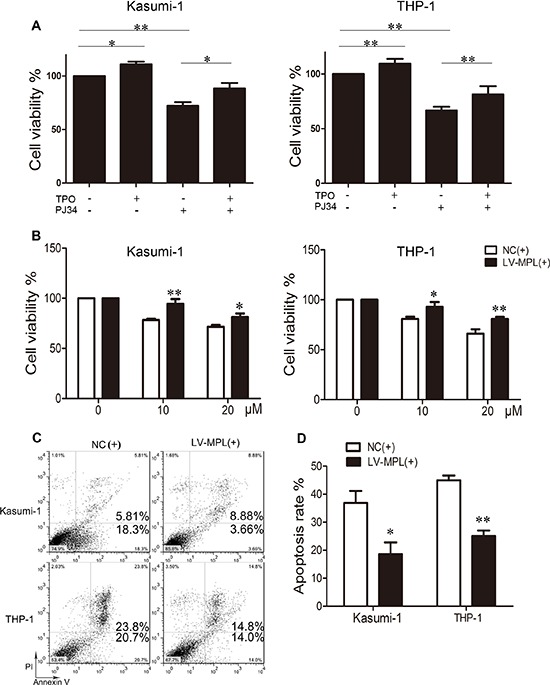
Enforced expression or activation of MPL partially rescues the effect of PARP-1 inhibition in AML cells **A.** Cell viability of Kasumi-1 and THP-1 cells with thrombopoietin (TPO) and/or PARP-1 inhibitor PJ34. *P < 0.05 and **P < 0.01, compared to control. **B.** Cell viability, **C.** flow cytometry of apoptosis and **D.** its quantification of AML cell lines overexpressing MPL and incubated with PARP-1 inhibitor PJ34 for 48 h. *P < 0.05 and **P < 0.01, LV-MPL vs. NC. Data represent the mean ± SEM.

To further determine whether MPL was functional downstream of PARP-1, MPL was physiologically activated by its ligand TPO or overexpressed by lentiviral transduction. TPO increased the proliferation of Kasumi-1 and THP-1 cells, as did MPL overexpression (*P* < 0.05 and *P* < 0.01 respectively; Fig. 7A). On the other hand, PARP-1 inhibition decreased cell viability (*P* < 0.01; Fig. 7A); however, the effect of PARP-1 inhibition diminished significantly when MPL was activated by TPO in Kasumi-1 and THP-1 cells (*P* < 0.05 and *P* < 0.01 respectively; Fig. 7A). Growth inhibition due to PARP-1 inhibition was rescued when both Kasumi-1 and THP-1 cells overexpressed MPL (*P* < 0.01 and *P* < 0.05; Fig. 7B). Moreover, the number of apoptotic cells was reduced significantly (Fig. 7C, 7D), effectively showing the opposite effects induced by MPL activation and PARP-1 inhibition. Therefore, PARP-1 may act on AML cells by regulating its downstream functional MPL gene directly or indirectly.

## DISCUSSION

Emerging evidence show that PARP-1 participates in a broad variety of cellular biological processes, such as transcriptional regulation [4] and tumor angiogenesis [28], independently of its classical DNA damage repair function. So far, the precise signaling mechanism by which PARP-1 aggravates the pathogenesis of AML is still unclear. In the current study, we focused on the function of PARP-1 and its potential downstream molecule MPL, which was revealed by microarray analysis. PARP-1 inhibition suppressed the proliferation, arrested the cell cycle, and induced apoptosis of AML cells *in vitro* and alleviated disease progression and prolonged survival in mice *in vivo*. Both PARP-1 and MPL expression were upregulated in BM of AML patients, with a significant positive correlation. MPL supported AML cell proliferation, and activation or overexpression of MPL partly reversed PARP-1 inhibition effect.

One of the distinguishing features of malignant tumor cells is infinite proliferation. The rapid replication and division of tumor cells is inevitably accompanied by increasing DNA damage. PARP-1, a nuclear protein involved in DNA repair, is usually overexpressed in tumor tissue [6–8, 29, 30]. Here, we confirmed that PARP-1 expression is higher in BM from AML patients than from healthy individuals. However, its expression did not differ among different subtypes of AML according to the French–American–British (FAB) classification (data not shown). It has been previously reported that PARP-1 supports tumor cell growth, and inhibitors of PARP-1 show potent antiproliferative and proapoptoic effects, holding promise for tumor therapy [8, 11, 13, 31]. In this study, both PARP-1 inhibitor PJ34 treatment and PARP-1 knockdown induced significant suppression of AML cell growth. Cell proliferation is dependent on the cell cycle proceeding, which is highly ordered and tightly regulated by cyclins, cyclin-dependent kinases (CDKs), and CDK inhibitors (CKIs) [32]. Indeed, PARP-1 inhibition induced a significant G2/M cell cycle arrest, and downregulation of both cyclin B1 and CDK1, whereas CKI P27 was upregulated. In addition, abnormal activation of the Akt and ERK1/2 pathways promotes survival and suppresses apoptosis of leukemic cells, which could be effective therapeutic targets [33, 34]. Here, we observed that PARP-1 inhibition decreased the phosphorylation of both Akt and ERK in AML Kasumi-1 and THP-1 cells, which led to an increased in cell apoptosis. Further evidence was the anti-apoptotic Bcl-2 and Bcl-xL reduced levels. PARP-1 deficiency or inhibition can have a beneficial outcome in tumor treatment [35, 36]. Accordingly, we studied an AML murine model that allowed us to evaluate PARP-1 effect on AML infiltration and disease progression by monitoring C1498-GFP cells *in vivo*. As compared with controls, PARP-1 inhibition indeed alleviated the disease progression, including decreasing cell infiltration, hepatosplenomegaly, and prolonging survival, for further evidence of PARP-1 inhibition potentiality in tumor therapy. Of note, we confirmed PARP-1 overexpression in AML, promoting cell survival and AML progression.

In discussing the role of PARP-1 in tumors, almost exclusive focus is given to its DNA repair activity, including PARP-1-dependent base excision repair pathways and synthetic lethality of homologous repair-deficient (e.g., BRCA1- or BRCA2-deficient) tumors [37]. Recently, more efforts are being made to identify its roles in modulating chromatin structure, regulation of gene transcription and protein stability [4, 38], participating in the inflammatory response, immunologic process, vascular plasticity and metastasis, and epithelial-mesenchymal transition (EMT) [12, 39–41]. Therefore, we performed a transcriptome analysis to explore the underlying molecular mechanisms. Differentially expressed genes with PARP-1 inhibition were mainly enriched in regulation of transcription, metabolic process, cell proliferation, apoptotic process, and phosphorylation, which supports PARP-1 function in inflammation, tumor, and metabolic disorders. These results provide a better understanding of some tumor-related genes regulated by PARP-1. Martínez-Bosch and colleagues proved that PARP-1 upregulates MDM2, VEGFR1, and MMP28, accelerating tumor proliferation and angiogenesis in pancreatic cancer [42]. Moreover, PARP-1 participates in the regulation of c-myc gene expression. Because c-myc regulates 10 to 15% of human genes, this interaction may be the basis of the pleiotropic physiological behavior of PARP-1 [43]. In our study, we tested several dysregulated genes associated with leukemia by microarray data analysis, including MPL, GADD45g, TOB1, SLA, and ETS1, and mainly focused on MPL, which was reduced by PARP-1 inhibition.

As the receptor of TPO, MPL is mainly expressed on the membrane of HSPCs, megakaryocytes, and thrombocytes. Physiologically, MPL plays an important role in the proliferation and differentiation of HSPCs and megakaryocytes, and in the maintenance of an appropriate number of platelets [44]. However, excessive activation of the TPO/MPL pathway is involved in leukemogenesis [45] as well as in chemotherapy resistance [24]. Recent studies showed that TPO/MPL/Bcl-xL or TPO/MPL/PI3K/Akt signaling pathways are essential for maintaining AML driven by AML1-ETO [22, 23]. Here, we demonstrate that MPL was highly expressed in clinical AML specimens, as previously described [46, 47]. In addition, PARP-1 and MPL expression were positively correlated in our clinical samples, which further suggest MPL as an important intermediary of PARP-1-mediated AML progress. This hypothesis was verified by activation of MPL, upon stimulation by its ligand TPO or lentivirus-mediated overexpression, where PARP-1 function was hindered. Accordingly, functional studies showed that the anti-proliferative and pro-apoptotic function of PARP-1 inhibition was partly reversed by MPL activation. These data suggested that PARP-1 takes effect at least in part by its ability to activate MPL. Nevertheless, the direct or indirect relationship between PARP-1 and MPL remains to be elucidated. MPL is well accepted as an oncogene, and gain-of-function or loss-of-function of MPL specifically showed significant growth promotion or inhibition, respectively. TPO is the classical ligand of MPL, activating its downstream molecular pathways, including PI3K/Akt and MAPK pathways [48, 49]. Our study revealed that Akt, ERK1/2, JNK, and p38 pathways were constitutively activated in AML; however, MPL knockdown inhibited only p-Akt and p-ERK levels, perhaps because of different biological characteristics of various cell types.

Currently, more and more gene mutations, aberrantly expressed genes, and microRNAs are identified in AML [50]. On one hand, these molecular disorders can be potential prognostic markers of risk stratification and choice of therapeutic regimens; and on the other hand, they may be effective targets for clinical treatment. In previous reports, PARP-1 is statistically considered as a prognostic marker in solid tumors, where high expression of PARP-1 associated with the worse outcomes [9, 10, 51]. We analyzed the relation between chemotherapeutic response of AML patients and initial PARP-1 expression. The results revealed that patients with high PARP-1 expression levels associated with a refractory response or died in a short time (Supplementary Fig. 2). From these refractory patients also emerged an overexpression of MPL, verifying its role of adverse prognostic factor [52]. Since chemotherapies remain the primary therapy for AML treatment; however generating more DNA damage, which can further activate PARP-1, and contributing to chemotherapy resistance. Inhibition of PARP-1 became important to oncotherapy. In recent years, encouraging results have been achieved by PARP-1 inhibitors in treating BRCA 1/2-related breast and ovarian cancers [53, 54], and here we observed that a PARP-1 inhibitor also alleviates AML tumor load and prolongs survival in mice. Additional studies are required to uncover the specific impact of PARP-1 on diagnosis, therapy, and prognosis.

**Figure 8:**
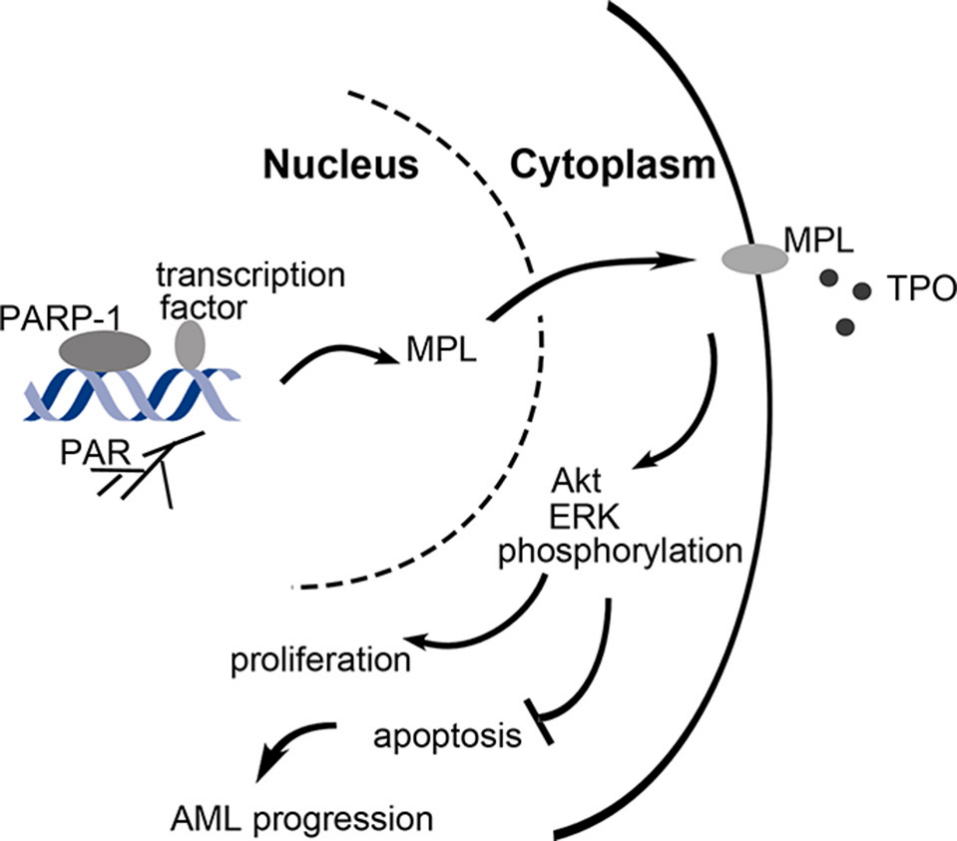
Schematic representation of the role of PARP-1 in the regulation of AML cell survival and proliferation PARP-1 is overexpressed in AML, which may be caused by DNA damage when excessive division and proliferation of tumor cells. As a transcriptional coregulator, PARP-1 upregulates MPL expression by polyADP-ribosylation, which further activates Akt and ERK1/2 pathways, and allows malignant cells to unlimited proliferation and escaping apoptosis.

Taken together, PARP-1 overexpression in AML possibly caused by DNA damage which were associated with excessive division and proliferation of tumor cells may activate MPL. Furthermore, Akt and ERK1/2 pathways were highly activated, endowing malignant cells with the ability of aberrant proliferation and escaping apoptosis (Fig. 8). Understanding this pathway provides important molecular insights into the underlying mechanisms of PARP-1 in AML. More in-depth investigations are needed to elucidate the potential mechanisms by which PARP-1 regulates downstream molecules.

## MATERIALS AND METHODS

### Reagents

PARP-1 inhibitor (PJ34) was purchased from Sigma-Aldrich (St. Louis, MO, USA), and TPO was purchased from 3SBIO (Shenyang, China). Primary antibodies for PARP-1 and β-actin were obtained from Santa Cruz Biotechnology (Santa Cruz, CA, USA) and for anti-poly(ADP-Ribose) (PAR) from BD Biosciences (NJ, USA). Antibodies against MPL was obtained from Abcam (Cambridge, MA, USA) and phosphorylated extracellular signal-regulated kinase 1/2 (p-ERK)/ERK, p-P38/P38 mitogen-activated protein kinase (MAPK), p-c-Jun N-terminal kinase (JNK)/JNK and p-Akt/Akt were all purchased from Cell Signaling Technology (Beverly, MA, USA).

### Cell lines

The C1498 murine AML cell line (ATCC) was cultured in complete Dulbecco’s Modified Eagle Medium (DMEM; Gibco, Carlsbad, CA, USA) supplemented with 10% fetal bovine serum (FBS; Gibco, Grand Island, NY, USA). C1498-GFP cells were produced by retroviral transduction and selected with puromycin (6 μg/mL) for 2 weeks. THP-1 and Kasumi-1 (Shanghai Institutes for Biological Sciences, China) cell lines that were derived from AML patients were cultured in RPMI-1640 medium (Gibco, Carlsbad, CA, USA) supplemented with 10% FBS and 1% penicillin-streptomycin. Cells were maintained in a humidified incubator at 37°C and 5% CO_2_.

### Patient samples

Bone marrow (BM) was collected from AML patients with a blast cell median of 75% (28–99%) among leucocytes and from healthy donors at Qilu Hospital, Shandong University. Mononuclear cells were obtained from BM by density-gradient centrifugation with Ficoll-Hypaque (Sigma-Aldrich, St. Louis, MO, USA). A written informed consent was obtained from all donors, and study protocols were approved by the medical ethics committee of the Affiliated Qilu Hospital of Shandong University (Jinan, China).

### Animal model and treatment

C57BL/6J male mice, from Jackson Laboratory (Bar Harbor, ME, USA), were housed in a specific pathogen-free animal care facility under a constant temperature (24°C) and regular light-dark cycles. Mice were given a normal diet and free access to water. C1498-GFP cells (1 × 10^6^)were resuspended in phosphate buffered saline (PBS; 100 μL) and injected into 6–8 weeks old mice via the tail vein. Experimental and control mice were intraperitoneally injected with PARP-1 inhibitor (10 mg/kg) or normal saline (NS) once a day for four weeks. Mice were observed closely for occurrence and development of disease. Blood was drawn from the venae angularis and resuspended in red blood cell lysis buffer to remove erythrocytes. The remaining cells were used to analyze GFP-positive cells by flow cytometry. Moreover, liver tissues were mechanically dissociated into a monoplast suspension for detection of leukemic cell infiltration by flow cytometry. Paraffinized sections of liver were stained with hematoxylin and eosin (H&E) staining. All experimental procedures were performed in accordance with the Guide for the Care and Use of Laboratory Animals published by the US National Institutes of Health and Shandong University.

### Microarray analysis

C1498 cells were treated with PARP-1 inhibitor or PBS, and total RNA was extracted for microarray assay. Differentially expressed genes were detected with the Affymetrix GeneChip Mouse Gene 1.0 ST Array (Affymetrix, CA, USA), and GO-Analysis and Pathway-Analysis was performed by Gminix (Shanghai). GEO accession number: GSE64220, http://www.ncbi.nlm.nih.gov/geo/query/acc.cgi?token=orezmycgvjqvpox&acc=GSE64220

### Proliferation assay

The cell viability of treated cells was evaluated by Cell Counting Kit-8 (CCK-8; DOJINDO, Japan). Briefly, cells were cultured in 96-well plates, seeded at 3–5 × 10^3^ per well. The CCK8 solution (10 μL) was added to each well and incubated at 37°C for 1 to 4 h. Absorbance was measured on a microplate reader (Bio-Rad) at 450 nm. Each sample was analyzed in triplicate.

### Apoptosis assay

Apoptosis was analyzed with FITC Annexin V Apoptosis Detection Kit I (BD Biosciences, NJ, USA). Cells were washed with PBS, resuspended in 100 μL binding buffer, labeled with 5 μL Annexin V-FITC and 5 μL PI, and incubated for 15 min in the dark. The binding buffer (400 μL) was added and the fluorescence was detected on a FACS Calibur (Becton Dickinson, CA, USA).

### Cell cycle analysis

Cells treated with 10 μM PARP-1 inhibitor for 24 h were harvested, fixed in ice-cold 75% ethanol at 4°C for 2 h, resuspended in PBS to rehydrate for 15 min, and collected and resuspended in DNA staining solution (MultiSciences Biotech, Hangzhou, China) for 30 min at room temperature. DNA content was analyzed in triplicate on FACS Calibur (Becton Dickinson, CA, USA). Cell cycle distribution was analyzed with the ModFit LT 3.1 software (Verity Software House, ME, USA).

### Quantitative real-time PCR

Total RNA from cell lines or patient BM samples was extracted in TRIzol reagent (Invitrogen, Carlsbad, CA, USA). RNA was reverse-transcribed into cDNA with the PrimeScript RT reagent kit with gDNA Eraser (TaKaRa, Dalian, China). qRT-PCR was carried out in a 20-μL reaction volume with SYBR Green PCR Master Mix (Toyobo, Osaka, Japan) on a real-time PCR thermocycler (IQ5 Real-Time PCR cycler, Bio-Rad). Relative expression was normalized to that of GAPDH. The primers for real-time quantification are listed in Table 3.

**Table 3:** Primer sets and genes included in the qRT-PCR analysis

Name	Forward primer (5′–3′)	Reverse primer (5′–3′)
PARP-1 (homo)	TCTGAGCTTCGGTGGGATGA	TTGGCATACTCTGCTGCAAAG
MPL (homo)	TTTCTCCCGAACATTTGAGG	GTGCAGCGGAAAGAAGAGAC
GAPDH (homo)	GGTGAAGGTCGGAGTCAACG	TGGGTGGAATCATATTGGAA
MPL (mus)	CTGTATGCCTACCGAGGAGAG	TCTGGTTGAGGGACACATTCTT
GADD45g (mus)	CTGCTGTGAGAACGACATTGA	CTCTCCTCGCAGAACAAACTG
TOB1 (mus)	ATATGAAGGGCACTGGTATCCT	GGATGCCTGCTCGATCACG
SLA (mus)	ATGGGGAATAGCATGAAATCCAC	GGAGATGGGTAGTCAGTCAGC
ETS1 (mus)	CCCTGGGTAAAGAATGCTTCC	GCTGATGAAGTAATCCGAGGTG
GAPDH (mus)	CATGGCCTTCCGTGTT CCTA	CCTGCTTCACCACCTTCTTGAT

### Western blot analysis

Cells were harvested by centrifugation and lysed in RIPA buffer (Beyotime, Haimen, China) supplemented with protease and phosphatase inhibitors (Beyotime) for 30 min for protein extraction. Samples were separated on 10% SDS-PAGE, transferred to polyvinylidene fluoride membranes or nitrocellulose membranes blocked with 5% non-fat milk for 1 to 2 h at room temperature, incubated with primary antibodies overnight at 4°C and with horseradish peroxidase-conjugated secondary antibodies for 2 h at room temperature. Signals were detected by chemiluminescence (Milipore, Billerica, USA). Expression levels were normalized to β-actin expression as the internal control, and quantification was performed with the ImageJ software.

### Statistical analysis

Data are presented as mean ± SEM. Results were compared by 2-tailed Student’s *t* test for 2 groups and one-way ANOVA for multiple groups with the SPSS 16.0 software (SPSS Inc., Chicago, IL). Correlation analysis was determined by Spearman’s test. *P* < 0.05 was considered statistically significant.

## SUPPLEMENTARY FIGURES


