# Supplementary material for: Inhibition of poly(ADP-ribose) polymerase 1 protects against acute myeloid leukemia by suppressing the myeloproliferative leukemia virus oncogene
Source: Oncotarget. 2015 Jul 25;6(29):27490–504. doi: 10.18632/oncotarget.4748 (PMC4695004; doi:10.18632/oncotarget.4748)
Supplement: Supplementary file 1 [file oncotarget-06-27490-s001.pdf]

SUPPLEMENTARY FIGURES

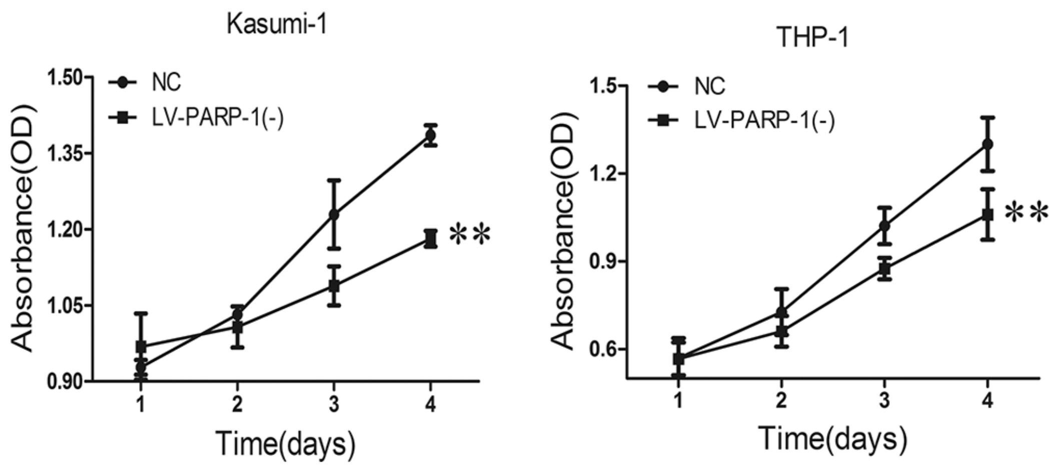

Supplementary Figure S1: Growth inhibition of AML cell lines by PARP-1 gene silencing. Cell viability of Kasumi-1 and THP-1 cells with PARP-1 knockdown or negative control (NC). $^{**}P < 0.01$, LV-PARP-1(-) vs. NC. Data represent the mean ± SEM.

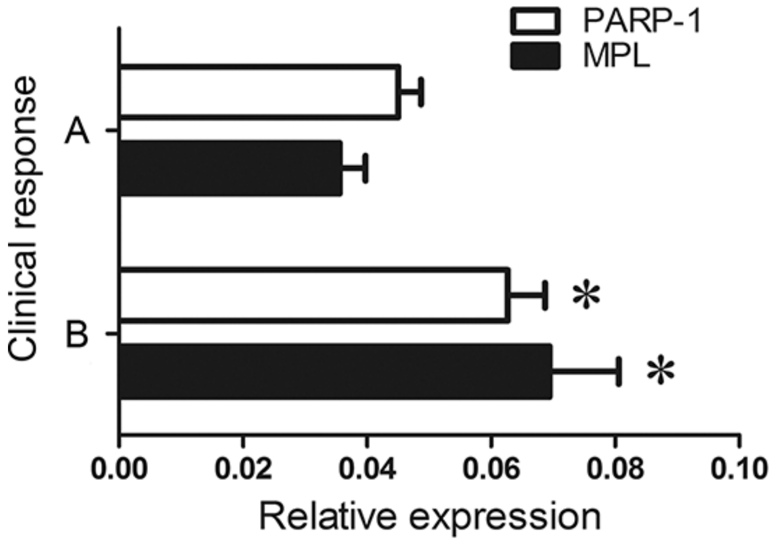

Supplementary Figure S2: Relationship between PARP-1 and MPL expression, and clinical responses in AML patients. Patients were divided into two groups according to their clinical response to chemotherapy. A: Achieving complete remission (CR) within two chemotherapy courses and sustaining CR. B: Not achieving CR within two chemotherapy courses. Patients with no follow-up were excluded. High expression levels of PARP-1 and MPL were associated with refractoriness. *$P < 0.05$. Data represent the mean ± SEM.
